# Taxonomic and phylogenetic analyses reveal two new *Comoclathris* taxa (Dothideomycetes, Pleosporaceae) from Inner Mongolia, China

**DOI:** 10.3897/mycokeys.129.179192

**Published:** 2026-02-27

**Authors:** Xing-Guo Tian, Tian-Ye Du, An-Dong Ma, Rajesh Jeewon, Samantha C. Karunarathna, Saowaluck Tibpromma, Dan-Feng Bao

**Affiliations:** 1 School of Food and Pharmaceutical Engineering, Guizhou Institute of Technology, Guiyang 550003, China Guizhou Key Laboratory of Agricultural Microbiology, Guizhou Academy of Agricultural Sciences Guiyang China; 2 Guizhou Key Laboratory of Agricultural Microbiology, Guizhou Academy of Agricultural Sciences, Guiyang 550009, China School of Pharmacy, Zunyi Medical University Zunyi China; 3 Yunnan International Joint Laboratory of Digital Conservation and Germplasm Innovation and Application of China-Laos Tea Resources, School of Tea and Coffee, Pu’er University, Pu’er 665000, China College of Biology and Food Engineering, Qujing Normal University Qujing China; 4 Department of Health Sciences, Faculty of Medicine and Health Sciences, University of Mauritius, Réduit 80837, Mauritius Kunming Institute of Botany, Chinese Academy of Sciences Kunming China; 5 Honghe Center for Mountain Futures, Kunming Institute of Botany, Chinese Academy of Sciences, Honghe County 654400, Yunnan, Kunming, China Faculty of Medicine and Health Sciences, University of Mauritius Réduit Mauritius; 6 School of Pharmacy, Zunyi Medical University, Zunyi, Guizhou Province 563000, China School of Food and Pharmaceutical Engineering, Guizhou Institute of Technology Guiyang China; 7 Center for Yunnan Plateau Biological Resources Protection and Utilization & Yunnan International Joint Laboratory of Fungal Sustainable Utilization in South and Southeast Asia, College of Biology and Food Engineering, Qujing Normal University, Qujing 655099, China School of Tea and Coffee, Pu’er University Pu’er China

**Keywords:** 2 new species, desert, Dothideomycetes, Pleosporales, saprobes

## Abstract

The Inner Mongolia Autonomous Region, located in northern China, represents a temperate semi-arid ecosystem dominated by steppe vegetation and characterized by rich, yet understudied, fungal diversity. During an investigation of microfungi associated with decaying plant materials in the desert regions of Inner Mongolia, two novel species of *Comoclathris* were collected and isolated. Detailed morphological examinations and multi-locus phylogenetic analyses based on ITS, LSU, SSU, and *rpb*2 sequence data support the establishment of these taxa as novel species: *Comoclathris desertica* and *C. xiangshawanensis*. These two species are described and illustrated herein, with particular attention to their differences from similar species with respect to ascomatal structures and ascospore morphology. The discovery of these new taxa expands the known diversity and distribution of *Comoclathris* in China and provides new insights into the saprobic fungal communities of semi-arid regions. This study also highlights the ecological and taxonomic significance of continued surveys on saprobic fungi in grassland and desert ecosystems of northern China.

## Introduction

*Comoclathris* Clem. is a genus of ascomycetous fungi belonging to Pleosporaceae Nitschke (Pleosporales, Dothideomycetes). The genus was established by Clements (1909), with *C. lanata* Clem. designated as the type species. Previously, the genus *Comoclathris* was placed in Diademaceae Shoemaker & C.E. Babc. (Shoemaker and Babcock 1992), but recent morphological and molecular phylogenetic analyses have supported its placement in Pleosporaceae (Zhang et al. 2012; Ariyawansa et al. 2015; Hyde et al. 2024). Currently, 52 epithets of *Comoclathris* are listed in Index Fungorum (2026), with molecular data available for only 21 species in NCBI. This genus is characterized by ascomata with circular lid-like openings, cylindrical to clavate asci, and muriform ascospores that are reddish-brown to dark brown and applanate (Mattoo et al. 2023). While most *Comoclathris* species are mainly saprobic on woody substrates, dead stems, and leaves of various plants or soil (Wanasinghe et al. 2015; Thambugala et al. 2017; Brahmanage et al. 2020; Crous et al. 2021), some are endophytes (González-Menéndez et al. 2018; Mattoo et al. 2023) and phytopathogens (Moral et al. 2017). *Comoclathris* is a widely distributed genus, found in many countries and regions, as detailed in recent publications (Mattoo et al. 2023; Xu et al. 2024). *Comoclathris* was first reported in China by Xu et al. (2024), who described two novel species from Jilin and Yunnan provinces.

The Inner Mongolia Autonomous Region is located in northern China and lies within a temperate semi-arid zone, with ecological types covering typical steppe, meadow steppe, desert steppe, as well as sandy land and forest-steppe transition zones (Han et al. 2009; Su et al. 2011; Yan et al. 2025). The vegetation of this region is dominated by herbaceous plants of the Asteraceae Bercht. & J.Presl, Fabaceae Lindl., and Poaceae (R. Brown) Barnhart families, and although steppe vegetation predominates, the types are relatively diverse rather than strictly monotypic (Fu et al. 2021; Wang et al. 2024). Despite this lack of vegetation diversity, fungi are important in these regions, as they may harbour new species with significant bioprospecting potential. Recent studies have shown that the fungal communities in Inner Mongolia exhibit considerable diversity. For example, analyses from 63 root samples during a study of ectomycorrhizal (EM) fungi revealed 288 operational taxonomic units (OTUs) spanning 31 lineages, indicating that fungal communities in forest/forest-edge environments may be much more diverse than anticipated (Wang et al. 2021). Further studies on arbuscular mycorrhizal (AM) fungi in grassland environments conducted by Wang et al. (2020) revealed 24 species spanning across eight genera. Despite increasing research on fungal diversity in Inner Mongolia Autonomous Region, detailed data and systematic taxonomic studies on saprobic fungi remain scarce. To date, only a few saprobic fungal novelties have been reported from Inner Mongolia, for example, *Diatrype betulaceicola* Z.E. Yang and Hai X. Ma, with polysporous asci described by Yang et al. (2022). The unique ecological conditions of the semi-arid grassland ecosystem in this region make saprobic fungi crucial in decomposing plant debris, cycling nutrients, and maintaining soil fertility. Therefore, studying the taxonomy of saprobic fungi is essential to unravel their hidden fungal diversity and potential applications.

Our ongoing studies in this region explore the diversity of saprobic ascomycetes inhabiting dead plant material in the desert regions of Inner Mongolia, specifically the Xiangshawan Scenic Area. During a survey of microfungi from decaying substrates, two species of *Comoclathris* were collected and examined. Morphological characteristics and phylogenetic analyses based on multi-locus sequence data (ITS, LSU, SSU, and *rpb*2) were employed to confirm their taxonomic placement within Pleosporaceae and support their establishment as novel species. This study provides new insights into the saprobic fungal diversity of northern China and expands the known distribution and species richness of the genus *Comoclathris*.

## Materials and methods

### Sample collection, morphological observation, single-spore isolation and preservation

**Figure 1.**
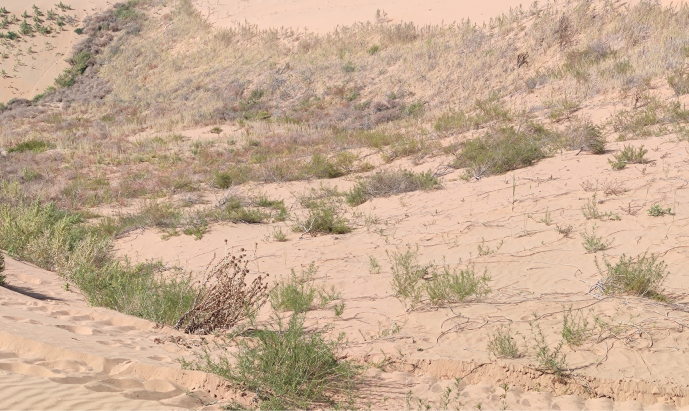
Vegetation and habitat in the desert area of Xiangshawan Scenic Area.

During a fungal resource survey in the desert areas of Inner Mongolia Autonomous Region, China (Fig. 1), dead fallen branches and stems with fungal fruiting bodies were collected. Important information was recorded during the collection process (collector, date, location, and habitat) (Rathnayaka et al. 2024). After that, the samples were placed in dry plastic bags for further observation and experimentation in the laboratory. Morphological structures were examined by using an OPTEC SZ650 dissecting stereomicroscope (Chongqing, China), and the microstructures of fungi were observed and photographed by using an OLYMPUS optical microscope (Tokyo, Japan) with an OLYMPUS DP74 (Tokyo, Japan) digital camera. Micro-morphological structures were measured in the Tarosoft ® Image Framework program v. 1.3. The photo plates were edited in Adobe Photoshop CS3 Extended version 22.0.0 software (Adobe Systems, California, USA).

Fungi were isolated and cultured using the single-spore isolation method described by Senanayake et al. (2020). The experiment was conducted under a sterile environment. After the work surface was disinfected with 75% alcohol, an alcohol lamp was lit. First, fresh fruiting bodies were observed and selected, then cut open with a sterile blade. Spores were picked up with a sterile needle, placed in sterile water and dispersed, and finally transferred to potato dextrose agar (PDA) plates (100 mL/plate) using a pipette. From 6 hours after inoculation to 48 hours, spores were checked promptly after germination with a sterile needle and then placed on a new PDA plate at 23–28 °C. Four germinated spores were transferred to each plate. After colonies formed, the morphology was observed to ensure consistency and confirm the results of the single-spore isolation experiment. The colonies were purified and used for molecular experiments, observed for sporulation, and photographed.

Specimens were deposited at Guizhou Medical University (GMB-W), China. Living cultures are deposited in the Guizhou Medical University Culture Collection (GMBCC), China. Facesoffungi (FoF) numbers were assigned as described by Jayasiri et al. (2015), and MycoBank (MB) numbers were assigned as outlined at https://www.mycobank.org/.

### DNA extraction, PCR amplification, and sequencing

Total genomic DNA was extracted from one-month-old fresh fungal mycelium (grown on PDA). The DNA Extraction Kit-BSC14S1 (BioFlux, Hangzhou, P.R. China) was used following the manufacturer’s instructions. Polymerase chain reactions (PCR) were carried out using the following primers: The internal transcribed spacer (ITS) region was amplified with the primers ITS4 and ITS5 (White et al. 1990), 28S nrRNA gene (LSU) was amplified by using the primers LR0R and LR5 (Vilgalys and Hester 1990), 18S ribosomal RNA (SSU) was amplified using the primers NS1 and NS4 (White et al. 1990), and the partial RNA polymerase II subunit (*rpb*2) region was amplified with primers fRPB2-5F and fRPB2-7cR (Liu et al. 1999). The PCR thermal cycle programs for ITS, LSU, and SSU were as follows: an initialization step of 94 °C for 3 min, followed by 35 cycles of 94 °C for 30 s, an annealing step at 55 °C for 50 s, an elongation step at 72 °C for 1 min and a final extension step of 72 °C for 10 min; and the PCR thermal cycle programs for *rpb*2 was as follows: an initialization step of 95 °C for 3 min, followed by 40 cycles of 95 °C for 50 s, an annealing step at 57 °C for 50 s, an elongation step at 72 °C for 90 s and a final extension step of 72 °C for 10 min (Du et al. 2025). The DNA amplification procedure was performed by PCR in a 25 μL reaction containing 12.5 μL 2xMaster Mix (mixture of Easy Taq TM DNA Polymerase, dNTPs, and optimized buffer (Beijing Trans Gen Biotech Co., Chaoyang District, Beijing, China)), 8.5 μL ddH_2_O, 2 μL of DNA template, and 1 μL of each forward and reverse primer (10 pM). Purification and sequencing of PCR products were carried out by Sangon Biotech Co., Kunming, China.

### Phylogenetic analyses

A combined dataset of ITS, LSU, SSU, and *rpb*2 was used for the phylogenetic analyses. The quality of the raw sequences was checked in BioEdit v.7.2.6.1 (Hall 1999), and the forward and reverse sequences were spliced with Geneious 9.1.8 (Kearse et al. 2012). Sequences obtained from this study were searched in the GenBank database (http://blast.ncbi.nlm.nih.gov/) using BLAST to identify the closely related taxa of our strains. The additional sequences included in the analyses were collected from previous publications (Mattoo et al. 2023; Xu et al. 2024) and downloaded from GenBank (Benson et al. 2013). Phylogenetic analyses were carried out with 41 sequences (Table 1). The FASTA file was used for constructing the Randomized Accelerated Maximum Likelihood (RAxML) and Bayesian Inference analyses (BI) was performed using the OFPT (Zeng et al. 2023) with the protocol. Then, the FASTA file was converted to PHYLIP and NEXUS formats in ALTER for RAxML and BI phylogenetic analyses, respectively (Glez-Peña et al. 2010).

**Table 1.** Taxa names, strain numbers, and corresponding GenBank accession numbers of the taxa included in the present study. Remarks: The newly generated sequences are indicated in bold, the superscript ^T^ indicates ex-type, and “—” indicates information unavailable.

Taxon name	Strain number	ITS	LSU	SSU	*rpb*2	References
*Comoclathris acuminata*	MCC9771^T^	MW205771	OQ547244	—	—	Mattoo et al. (2023)
*C. acuminata*	MN3-2019	OQ547243	OQ547245	—	—	Mattoo et al. (2023)
*C. ambigua*	CBS 366.52	KY940748	AY787937	—	KT216533	Woudenberg et al. (2017)
*C. antarctica*	WA0000074564^T^	MW040594	MW040597	—	—	Crous et al. (2021)
*C. arrhenatheri*	MFLUCC 15-0465^T^	KX965737	KY000647	KX986348	KX938346	Thambugala et al. (2017)
*C. arrhenatheri*	MFLUCC 15-0476	KY026595	KY000648	KX986349	—	Thambugala et al. (2017)
*C. clematidis*	CCMJ 13076^T^	OQ534243	OQ534239	OQ676454	OQ547800	Xu et al. (2024)
*C. clematidis*	CCMJ 13077	OQ534244	OQ534240	OQ676455	OQ547801	Xu et al. (2024)
*C. compressa*	CBS 156.53	—	KC584372	KC584630	KC584497	Woudenberg et al. (2013)
*C. compressa*	CBS 157.53	—	MH868679	KC584631	KC584498	Woudenberg et al. (2013)
***C. desertica***	**GMBCC2307^T^**	**PX660512**	**PX660516**	**PX660508**	**PX672979**	**This study**
***C. desertica***	**GMBCC2320**	**PX660513**	**PX660517**	**PX660509**	**PX672980**	**This study**
*C. europaeae*	MFLU 20-0391^T^	MT370396	MT370421	MT370367	MT729650	Brahmanage et al. (2020)
*C. flammulae*	MFLU 20-0397^T^	MT370397	MT370422	MT370368	MT729651	Brahmanage et al. (2020)
*C. flammulae*	MFLU 20-0399	MT370395	MT370420	MT370366	—	Brahmanage et al. (2020)
*C. galatellae*	MFLUCC 18-0773^T^	MN632549	MN632550	MN632551	—	Hongsanan et al. (2020)
*C. incompta*	CBS 467.76	—	GU238087	GU238220	KC584504	Aveskamp et al. (2010)
*C. incompta*	CH-16	KU973716	KU973729	—	—	Moral et al. (2017)
*C. italica*	MFLUCC 15-0073^T^	KX500109	—	—	—	Tibpromma et al. (2015)
*C. lini*	MFLUCC 14-0968^T^	KR049218	KR049219	KT210389	—	Wanasinghe et al. (2015)
*C. lini*	MFLUCC 14-0561	KT591614	KT591615	KT591616	—	Wanasinghe et al. (2015)
*C. lonicerae*	MFLU 20-0385^T^	MT370394	MT370419	MT370365	MT729649	Brahmanage et al. (2020)
*C. lonicerae*	MFLU 18-1236	OL744429	OL744433	OL744435	OL771441	Brahmanage et al. (2020)
*C. neimengguensis*	GMBCC2306^T^	PX314703	PX314701	PX314705	PX310456	Unpublished
*C. neimengguensis*	GMBCC2319	PX314704	PX314702	PX314706	PX310457	Unpublished
*C. permunda*	MFLUCC 14-0974	KY659561	KY659564	KY659568	—	Vu et al. (2019)
*C. pimpinellae*	MFLUCC 14-1159^T^	KU987665	KU987666	KU987667	—	Li et al. (2016)
*C. rosae*	MFLU 15-0203^T^	MG828876	MG828992	MG829103	MG829249	Wanasinghe et al. (2018)
*C. rosae*	MFLU 16-0234	MG828877	MG828993	MG829104	MG829250	Wanasinghe et al. (2018)
*C. rosarum*	MFLUCC 14-0962^T^	MG828878	MG828994	MG829105	MG829251	Wanasinghe et al. (2018)
*C. rosigena*	MFLU 16-0229^T^	MG828879	MG828995	MG829106	MG829252	Wanasinghe et al. (2018)
*C. sedi*	MFLUCC 13-0763^T^	KP334717	KP334707	KP334727	—	Ariyawansa et al. (2014)
*C. sedi*	MFLUCC 13-0817	KP334715	KP334705	KP334725	—	Ariyawansa et al. (2014)
*C. spartii*	MFLUCC 13-0214^T^	KM577159	KM577160	KM577161	—	Crous et al. (2014)
*C. typhicola*	CBS 602.72	MH860592	MH872288	—	—	Vu et al. (2019)
*C. xanthoceratis*	CCMJ 13078^T^	OQ534245	OQ534241	OQ676456	OQ547802	Xu et al. (2024)
*C. xanthoceratis*	CCMJ 13079	OQ534246	OQ534242	OQ676457	OQ547803	Xu et al. (2024)
***C. xiangshawanensis***	**GMBCC2305^T^**	**PX660510**	**PX660514**	**PX660506**	**PX672977**	**This study**
***C. xiangshawanensis***	**GMBCC2318**	**PX660511**	**PX660515**	**PX660507**	**PX672978**	**This study**
*Neocamarosporium betae*	CBS 523.66	FJ426981	MH870520	EU754080	KT389670	Aveskamp et al. (2009)
*N. calvescens*	CBS 246.79	MH861203	EU754131	EU754032	KC584500	Vu et al. (2019)

The CIPRES Science Gateway platform was used to carry out the RAxML and BI analyses (Miller et al. 2010). The RAxML tree, generated with 1,000 bootstrap replicates, was analyzed using RAxML-HPC2 on XSEDE (8.2.12) (Stamatakis et al. 2008; Stamatakis 2014) with the GTR+I+G model of evolution and bootstrap support. The BI tree was performed with MrBayes on XSEDE (3.2.7a) (Ronquist et al. 2012) by the Markov Chain Monte Carlo (MCMC) method to evaluate posterior probabilities (BYPP) (Richard and Lippmann 1991; Rannala and Yang 1996; Zhaxybayeva and Gogarten 2002). The best-fit nucleotide substitution models for each dataset were then selected using the Bayesian information criterion (BIC) from 22 common DNA substitution models with rate heterogeneity, as implemented in ModelFinder (Kalyaanamoorthy et al. 2017). The best model for ITS was TIM2e+I+G4, K2P+I for LSU, TPM2u+F+I for SSU, and TN+F+G4 for *rpb*2. Six simultaneous Markov chains were run for 2,000,000 generations, and a tree was sampled every 100^th^ generation. The phylogenetic tree was visualized in FigTree v.1.4.2 (Rambaut 2012) and edited in Microsoft PowerPoint 2021 and Adobe Photoshop CS3 Extended version 22.0.0 (Adobe Systems, California, USA). All newly generated sequences in this study were deposited in GenBank (https://www.ncbi.nlm.nih.gov/WebSub/?form=history&tool=genbank).

## Results

### Phylogenetic analyses

**Figure 2.**
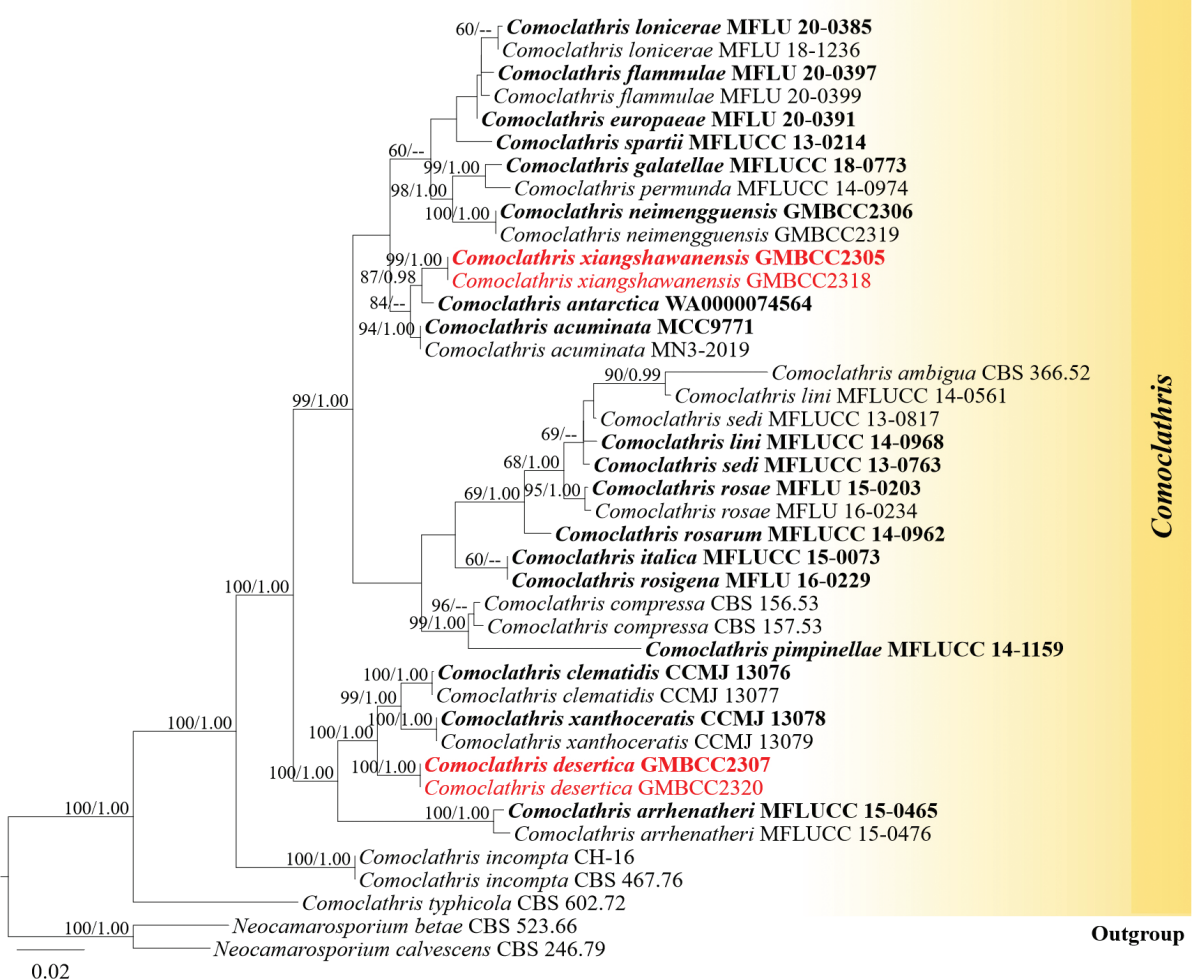
Maximum likelihood consensus tree inferred from the combined ITS, LSU, *rpb*2, and SSU multiple sequence alignments. Bootstrap support values for maximum likelihood (ML, first value) equal to or greater than 60% and Bayesian posterior probabilities from MCMC analyses (BYPP, second value) equal to or greater than 0.95 are given near the nodes. The scale bar indicates expected changes per site. The tree is rooted to *Neocamarosporium betae* (CBS 523.66) and *N. calvescens* (CBS 246.79). The new isolates are indicated in red, and the ex-type strains are in bold.

The phylogenetic trees obtained from RAxML and Bayesian Inference analyses were essentially similar. The RAxML analysis of the combined dataset yielded the best-scoring tree (Fig. 2), which consisted of 41 taxa, and the final alignment comprised 4029 characters, including gaps (ITS: 1–540, LSU: 541–1922, *rpb*2: 1923–3083, SSU: 3084–4029). The final ML optimization likelihood value was -14494.444982. The matrix contained 935 distinct alignment patterns, with 34.05% of characters undetermined or missing. Parameters for the GTR+I+G model of the combined ITS, LSU, *rpb*2, and SSU were as follows: estimated base frequencies A = 0.254158, C = 0.228977, G = 0.268187, T = 0.248678; substitution rates AC = 1.928002, AG = 4.366339, AT = 1.437220, CG = 1.029313, CT = 7.549462, GT = 1.0; proportion of invariable sites I = 0.641653; and gamma distribution shape parameter α = 0.725266. The final RAxML tree is shown in Fig. 2.

In our phylogenetic analyses, we obtained results consistent with those of recent publications by Mattoo et al. (2023) and Xu et al. (2024). Our new species, *Comoclathris desertica* (GMBCC2307 and GMBCC2320), was well separated from *C. clematidis* R. Xu, Phukhams. & Yu Li (CCMJ 13076 and CCMJ 13077) and *C. xanthoceratis* R. Xu, Phukhams. & Yu Li (CCMJ 13078 and CCMJ 13079) in a distinct lineage with 100% ML/1.00 PP statistical support; *Comoclathris xiangshawanensis* (GMBCC2305 and GMBCC2318) was well separated from *C. antarctica* Istel, J. Pawłowska & Wrzosek (WA0000074564, ex-type) with 87% ML/0.98 PP statistical support.

### Taxonomy

#### Comoclathris desertica

Taxon classificationFungiPleosporalesDiademaceae

X.G. Tian, T.Y. Du & D.F. Bao sp. nov.

74F9D643-66EA-5A91-8FAD-FF66B536FE4B

 MB861793

Facesoffungi Number: FoF19021

Fig. 3

##### Etymology.

Named after the desert habitat from where the holotype was collected.

##### Holotype.

GMB-W1518.

##### Description.

***Saprobic*** on dead branches of an unidentified wood. **Sexual morph**: ***Ascomata*** 90–200 × 70–190 μm (*x̄* = 115 × 116 μm, n = 5), solitary, scattered or aggregated, immersed, not obvious on the surface of the host, subglobose, black, ostiole not obvious. ***Peridium*** 10–30 μm wide, comprising thin-walled cells of ***textura angularis***, dark brown to black. ***Hamathecium*** comprising numerous, 2 μm wide, filamentous, septate, branched, cellular pseudoparaphyses, hyaline, embedded in a gelatinous matrix, extending above the asci. ***Asci*** 65–85 × 18–23 μm (*x̄* = 76 × 20 μm, n = 30), bitunicate, fissitunicate, 8-spored, cylindrical-clavate, short pedicellate, apically rounded. ***Ascospores*** 16–23 × 8–10 μm (*x̄* = 19 × 9 μm, n = 30), 1-seriate, slightly overlapping, fusiform, initially yellowish, becoming brown to dark brown, muriform, with 3 transversely septa and 1 vertical septum, consisting of 6 cells, upper end conical, lower end tapered gradually, apical cell mostly undivided, straight or slightly curved, smooth-walled, surrounded by a mucilaginous sheath. **Asexual morph**: Undetermined.

**Figure 3.**
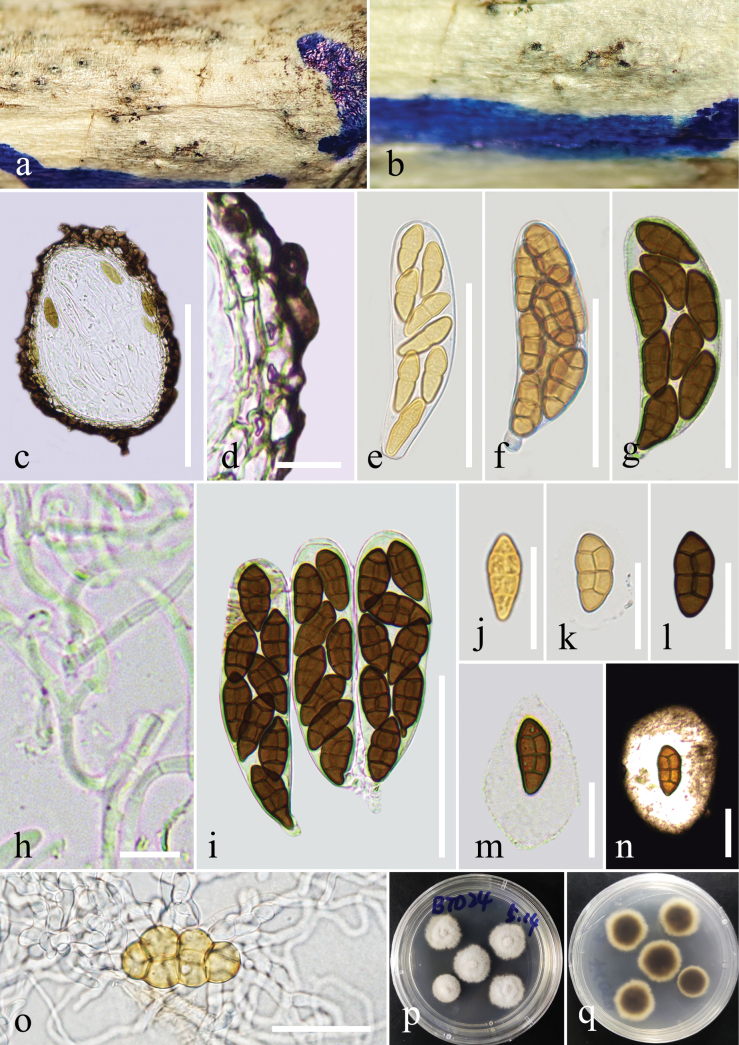
*Comoclathris desertica* (GMB-W1518, holotype). **a**, **b**. Appearance of ascomata on the substrate; **c**. Section through an ascoma; **d**. Peridium; **e-g, i**. Asci; **h**. Pseudoparaphyses; **j–n**. Ascospores (**m, n** surrounded by mucilaginous sheath); **o**. A germinated ascospore; **p, q**. Colonies on PDA from above and below after one week. Scale bars: 100 µm (**c**); 10 µm (**d, h**); 50 µm (**e-g, i**); 20 µm (**j–o**).

##### Culture characteristics.

Ascospores germinated on PDA within 12 h at 28 °C, and germ tubes were produced around spores. Colonies on PDA reached 2 cm diam. after one week at 28 °C. Colonies obverse: circular, white-cream to grey, with sparsely hairy edge; grey from below, the active hyphae at the edge are white.

##### Material examined.

China • Inner Mongolia Autonomous Region, Ordos City, Xiangshawan, on dead branches of an unidentified wood in the desert, 01 October 2023, J.Y. Zhang, BTD24 (GMB-W1518, holotype), ex-type, GMBCC2307, other living culture, GMBCC2320.

##### GenBank numbers.

GMBCC2307: ITS = PX660512, LSU = PX660516, SSU = PX660508, *rpb*2 = PX672979; GMBCC2320: ITS = PX660513, LSU = PX660517, SSU = PX660509, *rpb*2 = PX672980.

##### Notes.

*Comoclathris desertica* clustered with *C. clematidis* (CCMJ 13076, ex-type and CCMJ 13077) and *C. xanthoceratis* (CCMJ 13078, ex-type and CCMJ 13079) in the phylogenetic tree with 100% in ML and 1.00 in BYPP statistical support (Fig. 2). A nucleotide base pair differences between our new strain (GMBCC2307, ex-type) and *C. clematidis* (CCMJ 13076, ex-type) in ITS, LSU, SSU, and *rpb*2 (without gaps) showed a 2.70% (14/519 bp) difference in ITS, 1.33% (9/679 bp) difference in LSU, 0.32% (2/628 bp) difference in SSU, and 5.58% (47/842 bp) difference in *rpb*2. Between our new strain (GMBCC2307, ex-type) and *C. xanthoceratis* (CCMJ 13078, ex-type), there was a 2.35% (12/510 bp) difference in ITS, 1.18% (8/676 bp) difference in LSU, 0.32% (2/628 bp) difference in SSU, and 6% (52/866 bp) difference in *rpb*2.

Morphologically, *C. desertica* is similar to *C. clematidis* and *C. xanthoceratis*, both of which have ascospores with 3 transverse septa and a vertical septum, surrounded by a thick mucilaginous sheath. But *C. desertica* is different from *C. clematidis* and *C. xanthoceratis* in having immersed ascomata, and dark brown ascospores, 1-seriate, slightly overlapping, smooth-walled; while *C. clematidis* has immersed to erumpent ascomata, and brown ascospores, with verrucose or echinulate wall (Xu et al. 2024); while *C. xanthoceratis* has immersed to semi-immersed ascomata, and brown to dark brown ascospores, 1–2-seriate (Xu et al. 2024). And our collection displays comparatively smaller ascospores than these two species (viz., *C. desertica*: 19 × 9 μm, *C. clematidis*: 30 × 14 μm, *C. xanthoceratis*: 37 × 16 μm).

Therefore, *C. desertica* is introduced as a new species from China based on morphological and molecular evidence as recommended by Jeewon and Hyde (2016).

#### Comoclathris xiangshawanensis

Taxon classificationFungiPleosporalesDiademaceae

X.G. Tian, T.Y. Du & D.F. Bao sp. nov.

E45D5D8A-CF79-5DB2-A8EB-EBFFC06FD720

 MB861794

Facesoffungi Number: FoF19022

Fig. 4

##### Etymology.

Named after the type locality, “the desert region of Xiangshawan, China”.

##### Holotype.

GMB-W1526.

##### Description.

***Saprobic*** on dead branches of an unidentified wood. **Sexual morph**: ***Ascomata*** 100–200 × 90–200 μm (*x̄* = 132 × 118 μm, n = 10), solitary, scattered or aggregated into small groups, semi-immersed, subglobose, black, ostiole not obvious. ***Peridium*** 10–20 μm wide, comprising thin-walled cells of ***textura angularis***, dark brown to black. ***Hamathecium*** comprising numerous, 2 μm wide, filamentous, septate, branched, cellular pseudoparaphyses, hyaline, embedded in a gelatinous matrix, extending above the asci. ***Asci*** 65–85 × 15–23 μm (*x̄* = 73 × 21 μm, n = 30), bitunicate, fissitunicate, 8-spored, cylindrical-clavate, short pedicellate, apically rounded. ***Ascospores*** 17–23 × 7–11 μm (*x̄* = 19 × 8.5 μm, n = 30), 1–2-seriate, broadly fusiform, initially yellowish, becoming brown, muriform, with 5(–6) transversely septa and 1–2 vertical septa, consisting of 9–14 cells, with conical or obtuse ends, straight or slightly curved, smooth-walled, surrounded by a mucilaginous sheath. **Asexual morph**: Undetermined.

**Figure 4.**
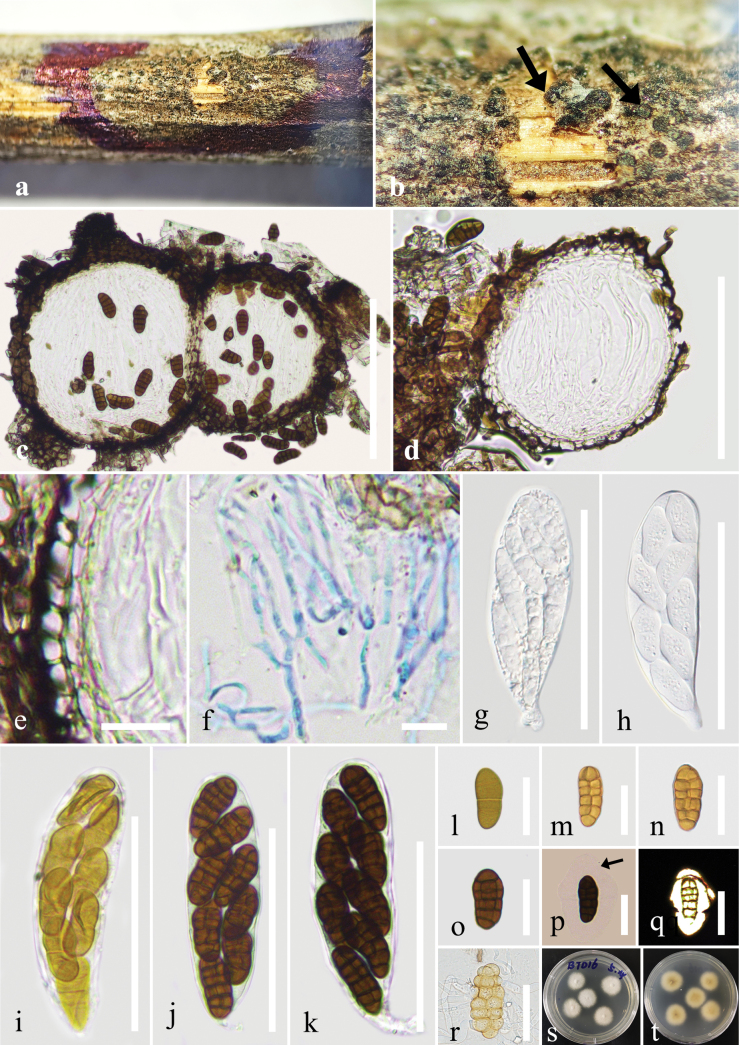
*Comoclathris xiangshawanensis* (GMB-W1526, holotype). **a**, **b**. Appearance of ascomata on the substrate (arrows indicate black ascomata); **c, d**. Section through ascomata; **e**. Peridium; **f**. Pseudoparaphyses; **g–k**. Asci; **l–q**. Ascospores (arrow indicates mucilaginous sheath); **r**. A germinated ascospore; **s, t**. Colonies on PDA from above and below after one week. Scale bars: 100 µm (**c, d**); 10 µm (**e, f**); 50 µm (**g–k**); 20 µm (**l–r**).

##### Culture characteristics.

Ascospores germinated on PDA within 12 h at 28 °C and germ tubes were produced around spores. Colonies on PDA reached 2 cm diam. after one week at 28 °C. Colonies obverse: circular, white-cream, with sparsely hairy edge; cream to light yellow from below.

##### Material examined.

China • Inner Mongolia Autonomous Region, Ordos City, Xiangshawan, on dead branches of an unidentified wood in the desert, 01 October 2023, J.Y. Zhang, BTD16 (GMB-W1526, holotype), ex-type, GMBCC2305, other living culture, GMBCC2318.

##### GenBank numbers.

GMBCC2305: ITS = PX660510, LSU = PX660514, SSU = PX660506, *rpb*2 = PX672977; GMBCC2318: ITS = PX660511, LSU = PX660515, SSU = PX660507, *rpb*2 = PX672978.

##### Notes.

*Comoclathris xiangshawanensis* clustered with *C. antarctica* (WA0000074564, ex-type) and *C. acuminata* Mattoo, Nonzom & A. Ghosh (MCC9771, ex-type and MN3-2019) in the phylogenetic tree (Fig. 2). A comparison of nucleotide base pair differences between our new strain (GMBCC2305, ex-type) and *C. antarctica* (WA0000074564, ex-type) in ITS and LSU, the result (without gaps) showed a 1.65% (8/484 bp) difference in ITS, 0.24% (2/818 bp) difference in LSU. Between our new strain (GMBCC2305, ex-type) and *C. acuminata* (MCC9771, ex-type) in ITS and LSU, the result (without gaps) showed a 1.86% (9/484 bp) difference in ITS, 0.25% (2/807 bp) difference in LSU (*C. antarctica* (WA0000074564, ex-type) and *C. acuminata* (MCC9771, ex-type) have no available SSU and *rpb*2 sequences in NCBI and hence cannot be compared).

Morphologically, *C. xiangshawanensis* differs from *C. antarctica* and *C. acuminata* in having brown ascospores with 5(–6) transversely septa and 1–2 vertical septa, consisting of 9–14 cells, surrounded by a mucilaginous sheath; while *C. antarctica* has ascospores with 6–8 transvers septa, consisting of 10–17 cells (Crous et al. 2021); while *C. acuminata* has dark brown ascospores, with 3–8 transverse septa, 3–5 vertical septa, consisting of 15–21 cells, with a beak-like extension (Mattoo et al. 2023). In addition, our collection is saprobic on dead branches in desert areas in China, while *C. antarctica* is isolated from soil sample in Antarctica, and *C. acuminata* was isolated as an endophyte from the stem of *Ephedra gerardiana* Wall. in India (Mattoo et al. 2023).

Therefore, *C. xiangshawanensis* is introduced as a new species from China based on morphological and molecular evidence. To provide a better overview of the different morphologies associated with different *Comoclathris* species, we also compiled ascospore morphology and other ecological data in a table (Table 2).

**Table 2.** Morphological characteristics of ascospore and other ecological data of *Comoclathris* species. NA: Information not available.

Species	Ascospore			Hosts	Collection locations	References
	Color	Septa	Sheath			
*Comoclathris acuminata*	Dark brown	3–8 transverse septa and 3–5 vertical septa	N/A	*Ephedra gerardiana*	India	Mattoo et al. (2023)
*Comoclathris antarctica*	Yellow to pale brown	6–8 transverse septa	N/A	Soil sample	Antarctica	Crous et al. (2021)
*Comoclathris arctica*	Mid to dark reddish-brown	6 transverse septa and 1 vertical septum	With a sheath 2–3 μm wide	*Festucae brachyphyllae*, *Puccinellia angustata*	Canada	Shoemaker and Babcock (1992)
*Comoclathris arrhenatheri*	Yellow to pale brown	4 transverse septa and 2–3 vertical septa	Surrounded by a thick mucilaginous sheath	*Arrhenatherum elatius*	Italy	Thambugala et al. (2017)
*Comoclathris bougainvilleae*	N/A	N/A	N/A	*Bougainvillea spectabilis*	India	Patil and Ramesh (1987)
*Comoclathris cichoriacearum*	N/A	N/A	N/A	*Hieracium villosum*	N/A	Nograsek (1990)
*Comoclathris clematidis*	Brown	3 transverse septa and 1 vertical septum	Surrounded by a thick mucilaginous sheath	*Clematis* sp.	China	Xu et al. (2024)
*Comoclathris compressa*	Dark reddish-brown	3 transverse septa and 1 vertical septum	With a uniform sheath 2–3 μm wide	*Agastache urticifolia*, *Agoseris glauca*, *Aquilegia leptocera*, *Arternisia pedatzjidi*, *Balsatnorrhiza deltoidea*, *Brickellia grandijlora*, *Coleosanthus grandijlorus*, *Castilleja miniata*, *Clematis hirsutissirna*, *Helianthella* sp., *Helianthus* sp., *Juncus balticus*, *Ligusticum purpureum*, *Lupinus parvijlorus*, *Penstemon* sp., *Polygonum alpina*, *Rudbeckia* sp., *Senecio thapsoides*, *Valeriana* sp., *Viguiera multijlora*, *Wyethia mollis*, *Wyethia* sp., *Xerophyllum tenux*	USA	Shoemaker and Babcock (1992)
*Comoclathris depressa*	Mid yellowish-brown	3 transverse septa and 1 vertical septum	With a sheath 2–3 μm wide	*Arabis* sp., *Lotnatiutn dissectum*	USA	Shoemaker and Babcock (1992)
*Comoclathris emodi*	Dark yellowish-brown	4 transverse septa and 1 vertical septum	With a sheath 1.5–4 μm wide	*Festucae lucidae*	India	Shoemaker and Babcock (1992)
*Comoclathris europaeae*	Brown	7 transverse septa and 1 vertical septum	Surrounded by amucilaginous sheath	*Olea europaea*	Italy	Brahmanage et al. (2020)
*Comoclathris extrema*	Dark reddish-brown	4 transverse septa and 1 vertical septum	With a sheath 2–3 μm wide	*Lini perenni*	India	Shoemaker and Babcock (1992)
*Comoclathris flammulae*	Dark brown	6 transverse septa and 1–2 vertical septa	Mucilaginous sheath	*Clematis flammula*, *Colutea arborescens*	Italy	Brahmanage et al. (2020)
*Comoclathris galatellae*	Brown or pale brown	2–4 transverse septa and 1–2 vertical septa	Without sheath	*Galatella villosa*	Russia, Ukraine	Hongsanan et al. (2020)
*Comoclathris harperi*	Mid reddish-brown	7 transverse septa and 1 vertical septum	With a sheath 1–2 μm wide	*Carex rupestris*, *Hierochloe alpina*	Sweden	Shoemaker and Babcock (1992)
*Comoclathris incompta*	N/A	N/A	N/A	*Olea europaea*	Greece, Italy	Ariyawansa et al. (2015)
*Comoclathris indica*	N/A	N/A	N/A	*Arachis hypogaea*	India	Patil and Pawar (1987)
*Comoclathris italica*	Brown	6–8 transverse septa and 1–2 vertical septa	Mucilaginous sheath	*Dactylis glomerata*	Italy	Thambugala et al. (2017)
*Comoclathris lanata*	Brown to reddish-brown	4–5 transverse septa and 1–2 vertical septa	Surrounded by a distinct, hyaline, mucilaginous 3–8 μm wide sheath	*Leptotaenia multifida*	USA	Ariyawansa et al. (2015)
*Comoclathris lini*	yellowish-brown	3–5 transverse septa and 1–2 vertical septa	With a thick mucilaginous sheath	*Linum* sp.	Italy	Wanasinghe et al. (2015)
*Comoclathris lonicerae*	yellowish-brown	3–5 transverse septa and 1–2 vertical septa	With a thick mucilaginous sheath	*Lonicera* sp.	Italy	Brahmanage et al. (2020)
*Comoclathris magna*	Dark reddish-brown	3 transverse septa and 1 vertical septum	With a sheath 2–4 μm wide	*Anemone occidentalis*, *Chlorogalum pomeridianum*, *Helianthella californica*, *Linum perenne*, *Lomatium nudicaule*, *Penstemon gracilentus*, *Sphenosciadium capitellatum*, umbellifer	USA	Shoemaker and Babcock (1992)
*Comoclathris miliarakisii*	N/A	N/A	N/A	*Sclerochorton junceum*	Greece	Petrak (1938)
*Comoclathris persica*	Mid reddish-brown	4 transverse septa and 1 vertical septum	With a uniform sheath 3–4 μm wide	*Dianthus orientalis*	Iran	Shoemaker and Babcock (1992)
*Comoclathris pimpinellae*	Yellow to light brown	3 transverse septa and 2 vertical septa	Surrounded by a thick, hyaline, a mucilaginous sheath	*Pimpinella tragium*	Russia	Li et al. (2016)
*Comoclathris platysporioides*	Dark reddish-brown	5 transverse septa and 1 vertical septum	With a uniform sheath, 1–2 μm wide	*Selaginella wrightii*	USA	Shoemaker and Babcock (1992)
*Comoclathris pseudoverruculosa*	N/A	N/A	N/A	*Carex firma*	N/A	Nograsek (1990)
*Comoclathris pyrenophoroides*	N/A	N/A	N/A	*Festuca* and *Bromus*	Italy	Checa (1998)
*Comoclathris quadriseptata*	Mid reddish-brown	4 transverse septa and 1 vertical septum	With a uniform sheath 1–2 μm wide	*Agropyron divergens*, *Avena pratensis*, *Dianthus orientalis*, *Matthiola* sp., *Poaceae*, *Sporobolus cryptandrus*	Canada	Shoemaker and Babcock (1992)
*Comoclathris rosae*	Pale brown	4–7 transverse septa and 1–2 vertical septa	Surrounded by a thick mucilaginous sheath	*Rosa canina*	Italy	Wanasinghe et al. (2018)
*Comoclathris rosarum*	Brown	6–7 transverse septa and 2–4 vertical septa	Surrounded by a thick mucilaginous sheath	*Rosa canina*	Italy	Wanasinghe et al. (2018)
*Comoclathris rosigena*	Pale brown	5–7 transverse septa and 1 vertical septum	Surrounded by a thick mucilaginous sheath	*Rosa canina*	Italy	Wanasinghe et al. (2018)
*Comoclathris salsolae*	Mid yellowish-brown	4 transverse septa and 1 vertical septum	With a uniform sheath 2–3 μm wide	*lpomoealeptoplzylla*, *Salsola* sp.	USA	Shoemaker and Babcock (1992)
*Comoclathris sedi*	Brown to reddish-brown	4–5 transverse septa and 1–2 vertical septa	Surrounded by a distinct, hyaline, mucilaginous 5–9 μm wide sheath	*Clematis vitalba*, *Rosa* sp., *Sedum* sp.	Italy	Ariyawansa et al. (2015)
*Comoclathris sisyrinchii*	Mid yellowish-brown	3 transverse septa and 1 vertical septum	N/A	*Carpha alina*, *Lomatium californicuin*, *Sisyrinchium junceum*, *Sisyrinchium middletoni*	USA	Shoemaker and Babcock (1992)
*Comoclathris sororia*	Dark reddish-brown	3 transverse septa and 1 vertical septum	N/A	*Dianthus orientalis*	Turkey	Shoemaker and Babcock (1992)
*Comoclathris spartii*	Yellow to pale brown	Muriform	Surrounded by a mucilaginous sheath	*Spartium junceum*	Italy	Crous et al. (2014)
*Comoclathris subdeflectens*	N/A	N/A	N/A	*Ranunculus montanus*	N/A	Nograsek (1990)
*Comoclathris typhicola*	Brown	3 transverse septa and 1 vertical septum	N/A	*Typha latifolia*, *Typha angustifolia*	Austria, Denmark, England, Germany, India, Iran, Netherlands, Pakistan, Poland, Portugal, Russia, Switzerland, and USA	Cooke (1872); Webster and Lucas (1959); Shoemaker and Babcock (1992); Boerema et al. (2004); Ahmadpour et al. (2024)
*Comoclathris utahensis*	Dark reddish-brown	3 transverse septa and 1 vertical septum	With a sheath 1–3 μm wide	*Arternisia scopulorurn*, *Brickelliaun bellata*, *Castilleja rniniata*, *Eupatoriurn occidentale*, *Linurn perenne*, *Linum perenne* L., *Polygonurn arnphibiurn*, *Senecio* sp., *Silene lnontana*	USA	Shoemaker and Babcock (1992)
*Comoclathris verrucosa*	Dark reddish-brown	3 transverse septa and 1 vertical septum	With a uniform sheath 2–3 μm wide	*Juncus balticus*	USA	Shoemaker and Babcock (1992)
*Comoclathris verruculosa*	Mid reddish-brown	5 transverse septa and 1 vertical septum	With a uniform sheath 1.5–3.5 μm wide	*Deschampsia caespitosa*, *Poa glauca*	Finland, Norway, Switzerland	Shoemaker and Babcock (1992)
*Comoclathris wehmeyeri*	N/A	N/A	N/A	*Crossandra infundibuliformis*	India	Pande (1980)
*Comoclathris xanthoceratis*	Brown to dark brown	3 transverse septa and 1 vertical septum	Surrounded by a thick mucilaginous sheath	*Xanthoceras sorbifolium*	China	Xu et al. (2024)
*Comoclathris xerophila*	Mid reddish-brown	3 transverse septa and 1 vertical septum	N/A	*Scleropogon brevifolius*	Argentina	Shoemaker and Babcock (1992)

## Discussion

*Comoclathris* is a widely distributed genus, recorded on all five continents viz. Asia, Europe, Africa, the Americas, and Antarctica (Mattoo et al. 2023; Xu et al. 2024). Recent studies in China and other regions have shown that *Comoclathris* species are distributed in a variety of environments and substrates, from humid forests to semi-arid deserts (Mattoo et al. 2023; Xu et al. 2024; this study), suggesting that their diversity and ecological adaptability may be underestimated. The discovery of *C. desertica* and *C. xiangshawanensis* in desert wood in Inner Mongolia further expands the known distribution of this genus, providing valuable data for the global taxonomic system of *Comoclathris*. Furthermore, the thick, gelatinous sheaths enclosing the ascospores of these two species highlight the adaptability to extremely arid environments of these species. This is in line with the general characteristics of this genus, with obvious mucilaginous sheaths surrounding the ascospores. Based on morphological information currently available for this genus (Table 2), most species have mucilaginous sheaths, with only seven species having no record of mucilaginous sheaths (viz., *C. acuminata*, *C. antarctica*, *C. galatellae* D. Pem, Bulgakov & K.D. Hyde, *C. sisyrinchii* (Speg.) Shoemaker & C.E. Babc., *C. sororia* (Bubák) Shoemaker & C.E. Babc., *C. typhicola* (Cooke) H.A. Ariy. & K.D. Hyde, and *C. xerophila* (Speg.) Shoemaker & C.E. Babc.) (Cooke 1872; Shoemaker and Babcock 1992; Hongsanan et al. 2020; Crous et al. 2021; Mattoo et al. 2023).

Although 52 epithets are listed in Index Fungorum (2026), only 21 species have molecular data in NCBI, and the naming of many historical species is based solely on morphological characteristics. The lack of DNA sequences, type specimen revisions, and living cultures hinder phylogenetic analysis and species delineation. Therefore, future work should collect more specimens and supplement them with molecular data of extant species to clarify species boundaries and evolutionary relationships.

Inner Mongolia is a typical semi-arid region where long-term grazing and desertification have led to the degradation of grasslands and soils. Fungi play a crucial role in the decomposition of organic matter and nutrient cycling in fragile ecosystems, but the diversity of saprobic fungi remains poorly understood. The discovery of two new *Comoclathris* species in the Xiangshawan desert demonstrates that even degraded or arid habitats can foster rich and unique fungal communities. These fungal groups reflect the potential ecological resilience and adaptive evolution of microfungi under extreme temperature and humidity fluctuations. This study not only enriches the known fungal diversity in northern China but also highlights the importance of ongoing mycological surveys in arid grassland and desert ecosystems for taxonomic progress and ecological conservation.

## Supplementary Material

XML Treatment for Comoclathris desertica

XML Treatment for Comoclathris xiangshawanensis

## Additional information

### Use of AI

No use of AI was reported.

### Author ORCIDs

Xing-Guo Tian https://orcid.org/0000-0002-8124-1281

Tian-Ye Du https://orcid.org/0000-0003-2105-1803

An-Dong Ma https://orcid.org/0009-0005-5981-3182

Rajesh Jeewon https://orcid.org/0000-0002-8563-957X

Samantha C. Karunarathna https://orcid.org/0000-0001-7080-0781

Saowaluck Tibpromma https://orcid.org/0000-0002-4706-6547

Dan-Feng Bao https://orcid.org/0000-0002-5697-4280

### Data availability

All of the data that support the findings of this study are available in the main text.

## References

[B1] Ahmadpour A, Ghosta Y, Alavi F, Alavi Z, Heidarian Z (2024) *Comoclathris typhicola*, a new species for the funga of Iran. Mycologia Iranica 11(1): 111–116. 10.3390/jof11030225

[B2] Ariyawansa HA, Phookamsak R, Tibpromma S, Kang JC, Hyde KD (2014) A molecular and morphological reassessment of Diademaceae. The Scientific World Journal 675348: 1–11. 10.1155/2014/675348 PMC3913511 24526916

[B3] Ariyawansa HA, Thambugala KM, Manamgoda DS, Jayawardena RS, Camporesi E, Boonmee S, Wanasinghe DN, Phookamsak R, Hongsanan S, Singtripop C, Chukeatirot E, Kang JC, Jones EBG, Hyde KD (2015) Towards a natural classification and backbone tree for Pleosporaceae. Fungal Diversity 71: 85–139. 10.1007/s13225-015-0323-z

[B4] Aveskamp MM, Verkley GJ, de Gruyter J, Murace MA, Perelló A, Woudenberg JH, Groenewald JZ, Crous PW (2009) DNA phylogeny reveals polyphyly of *Phoma* section *Peyronellaea* and multiple taxonomic novelties. Mycologia 101(3): 363–382. 10.3852/08-199 19537209

[B5] Aveskamp MM, Gruyter JD, Woudenberg JHC, Verkley GJM, Crous PW (2010) Highlights of the Didymellaceae: A polyphasic approach to characterise *Phoma* and related pleosporalean genera. Studies in Mycology 65(65): 1–60. 10.3114/sim.2010.65.01 PMC2836210 20502538

[B6] Benson DA, Cavanaugh M, Clark K, Karsch-Mizrachi I, Lipman DJ, Ostell J, Sayers EW (2013) GenBank. Nucleic Acids Research 41(D1): D36–D42. 10.1093/nar/gks1195 PMC3531190 23193287

[B7] Boerema GH, De Gruyter J, Noordeloos ME, Hamers MEC (2004) *Phoma* identification manual: differentiation of specific and infra-specific taxa in culture. CABI Publishing. 10.1079/9780851997438.0000

[B8] Brahmanage RS, Dayarathne MC, Wanasinghe DN, Thambugala KM, Jeewon R, Chethana KT, Samarakoon MC, Tennakoon DS, De Silva NI, Camporesi E, Raza M (2020) Taxonomic novelties of saprobic Pleosporales from selected dicotyledons and grasses. Mycosphere: Journal of Fungal Biology 11(1): 2481–2541. 10.5943/mycosphere/11/1/15

[B9] Checa J (1998) Annotated list of the pleosporacean fungi and related genera reported from the Iberian Peninsula and Balearic Islands. Mycotaxon 68: 205–249. 10.5962/p.415504

[B10] Clements FE (1909) The genera of fungi. The HW Wilson Company, 244 pp. 10.5962/bhl.title.54501

[B11] Cooke MC (1872) Grevillea Vol. 5, Issue 35. Williams and Norgate, 121. https://www.biodiversitylibrary.org/page/48315354

[B12] Crous PW, Wingfield MJ, Schumacher RK, Summerell BA, Giraldo A, Gené J, Guarro J, Wanasinghe DN, Hyde KD, Camporesi E, Gareth Jones EB, Thambugala KM, Malysheva EF, Malysheva VF, Acharya K, Álvarez J, Alvarado P, Assefa A, Barnes CW, Bartlett JS, Blanchette RA, Burgess TI, Carlavilla JR, Coetzee MPA, Damm U, Decock CA, den Breeÿen A, de Vries B, Dutta AK, Holdom DG, Latham SR, Manjón JL, Marincowitz S, Mirabolfathy M, Moreno G, Nakashima C, Papizadeh M, Fazeli SAS, Amoozegar MA, Romberg MK, Shivas RG, Stalpers JA, Stielow B, Stukely MJC, Swart WJ, Tan YP, van der Bank M, Wood AR, Zhang Y, Groenewald JZ (2014) Fungal Planet Description Sheets: 281–319. Persoonia 33: 212–289. 10.3767/003158514X685680 PMC4312934 25737601

[B13] Crous PW, Cowan DA, Maggs‐Kölling G, Yilmaz N, Thangavel R, Wingfield MJ, Noordeloos ME, Dima B, Brandrud TE, Jansen GM, Morozova OV, Vila J, Shivas RG, Tan YP, Bishop‐Hurley S, Lacey E, Marney TS, Larsson E, Le Floch G, Lombard L, Nodet P, Hubka V, Alvarado P, Berraf‐Tebbal A, Reyes JD, Delgado G, Eichmeier A, Jordal JB, Kachalkin AV, Kubátová A, Maciá‐Vicente JG, Malysheva EF, Papp V, Rajeshkumar KC, Sharma A, Spetik M, Szabóová D, Tomashevskaya MA, Abad JA, Abad ZG, Alexandrova AV, Anand G, Arenas F, Ashtekar N, Balashov S, Bañares Á, Baroncelli R, Bera I, Biketova AY, Blomquist CL, Boekhout T, Boertmann D, Bulyonkova TM, Burgess TI, Carnegie AJ, Cobo‐Diaz JF, Corriol G, Cunnington JH, da Cruz MO, Damm U, Davoodian N, de A Santiago ALCM, Dearnaley J, de Freitas LWS, Dhileepan K, Dimitrov R, Di Piazza S, Fatima S, Fuljer F, Galera H, Ghosh A, Giraldo A, Glushakova AM, Gorczak M, Gouliamova DE, Gramaje D, Groenewald M, Gunsch CK, Gutiérrez A, Holdom D, Houbraken J, Ismailov AB, Istel Ł, Iturriaga T, Jeppson M, Jurjević Ž, Kalinina LB, Kapitonov VI, Kautmanova I, Khalid AN, Kiran M, Kiss L, Kovács Á, Kurose D, Kusan I, Lad S, Læssøe T, Lee HB, Luangsa‐ard JJ, Lynch M, Mahamedi AE, Malysheva VF, Mateos A, Matočec N, Mešić A, Miller AN, Mongkolsamrit S, Moreno G, Morte A, Mostowfizadeh‐Ghalamfarsa R, Naseer A, Navarro‐Ródenas A, Nguyen TTT, Noisripoom W, Ntandu JE, Nuytinck J, Ostrý V, Pankratov TA, Pawłowska J, Pecenka J, Pham THG, Polhorský A, Posta A, Raudabaugh DB, Reschke K, Rodríguez A, Romero M, Rooney‐Latham S, Roux J, Sandoval‐Denis M, Smith MTH, Steinrucken TV, Svetasheva TY, Tkalčec Z, van der Linde EJ, vd Vegte M, Vauras J, Verbeken A, Visagie CM, Vitelli JS, Volobuev SV, Weill A, Wrzosek M, Zmitrovich IV, Zvyagina EA, Groenewald JZ (2021) Fungal Planet description sheets: 1182–1283. Persoonia 46: 313–528. 10.3767/persoonia.2021.46.11 PMC9311394 35935893

[B14] Du TY, Karunarathna SC, Hyde KD, Nilthong S, Mapook A, Dai DQ, Rajeshkumar KC, Elgorban AM, Han LS, Wang H-H, Tibpromma S (2025) New *Aquilariomyces* and *Mangifericomes* species (Pleosporales, Ascomycota) from *Aquilaria* spp. in China. MycoKeys 112: 103–125. 10.3897/mycokeys.112.139831 PMC11747782 39839667

[B15] Fu Z, Wang F, Lu Z, Zhang M, Zhang L, Hao W, Zhao L, Jiang Y, Gao B, Chen R, Wang B (2021) Community differentiation and ecological influencing factors along environmental gradients: Evidence from 1200 km belt transect across Inner Mongolia grassland, China. Sustainability 14(1): 361. 10.3390/su14010361

[B16] Glez-Peña D, Gómez‐Blanco D, Reboiro‐Jato M, Fdez‐Riverola F, Posada D (2010) ALTER, program‐oriented conversion of DNA and protein alignments. Nucleic Acids Research 38: W14–18. 10.1093/nar/gkq321 PMC2896128 20439312

[B17] González-Menéndez V, Crespo G, De Pedro N, Diaz C, Martín J, Serrano R, Mackenzie TA, Justicia C, González-Tejero MR, Casares M, Vicente F (2018) Fungal endophytes from arid areas of Andalusia: High potential sources for antifungal and antitumoral agents. Scientific Reports 8(1): 1–13. 10.1038/s41598-018-28192-5 PMC6021435 29950656

[B18] Hall TA (1999) BioEdit: A user-friendly biological sequence alignment editor and analysis program for Windows 95/98/NT. Nucleic Acids Symposium Series 41: 95–98. 10.1093/bib/bbx108

[B19] Han X, Owens K, Wu XB, Wu J, Huang J (2009) The grasslands of Inner Mongolia: A special feature. Rangeland Ecology and Management 62(4): 303–304. 10.2111/09-002.1

[B20] Hongsanan S, Hyde KD, Phookamsak R, Wanasinghe DN, McKenzie EHC, Sarma VV, Boonmee S, Lücking R, Pem D, Bhat JD, Liu N, Tennakoon DS, Karunarathna A, Jiang SH, Jones EBG, Phillips AJL, Manawasinghe I, Tibpromma S, Jayasiri SC, Sandamali D, Jayawardena RS, Wijayawardene NN, Ekanayaka AH, Jeewon R, Lu YZ, Dissanayake AJ, Zeng XY, Luo ZL, Tian Q, Phukhamsakda C, Thambugala KM, Dai DQ, Chethana TKW, Ertz D, Doilom M, Liu JK, Pérez-Ortega S, Suija A, Senwanna C, Wijesinghe SN, Konta S, Niranjan M, Zhang SN, Ariyawansa HA, Jiang HB, Zhang JF, de Silva NI, Thiyagaraja V, Zhang H, Bezerra JDP, Miranda-Gonzáles R, Aptroot A, Kashiwadani H, Harishchandra D, Aluthmuhandiram JVS, Abeywickrama PD, Bao DF, Devadatha B, Wu HX, Moon KH, Gueidan C, Schumm F, Bundhun D, Mapook A, Monkai J, Chomnunti P, Samarakoon MC, Suetrong S, Chaiwan N, Dayarathne MC, Jing Y, Rathnayaka AR, Bhunjun CS, Xu JC, Zheng JS, Liu G, Feng Y, Xie N (2020) Refined families of Dothideomycetes: Dothideomycetidae and Pleosporomycetidae. Mycosphere : Journal of Fungal Biology 11: 1553–2107. 10.5943/mycosphere/11/1/13

[B21] Hyde KD, Noorabadi MT, Thiyagaraja V, He MQ, Johnston PR, Wijesinghe SN, Armand A, Biketova AY, Chethana KWT, Erdoğdu M, Ge ZW, Groenewald JZ, Hongsanan S, Kušan I, Leontyev DV, Li DW, Lin CG, Liu NG, Maharachchikumbura SSN, Matočec N, May TW, McKenzie EHC, Mešić A, Perera RH, Phukhamsakda C, Piątek M, Samarakoon MC, Selcuk F, Senanayake IC, Tanney JB, Tian Q, Vizzini A, Wanasinghe DN, Wannasawang N, Wijayawardene NN, Zhao RL, Abdel-Wahab MA, Abdollahzadeh J, Abeywickrama PD, et al. (2024) The 2024 Outline of Fungi and fungus-like taxa. Mycosphere : Journal of Fungal Biology 15(1): 5146–6239. 10.5943/mycosphere/15/1/25

[B22] Index Fungorum (2026) Index Fungorum. http://www.indexfungorum.org/Names/Names.asp [accessed 15 January 2026]

[B23] Jayasiri SC, Hyde KD, Ariyawansa HA, Bhat J, Buyck B, Cai L, Dai YC, Abd‐Elsalam KA, Ertz D, Hidayat I, Jeewon R, Jones EBG, Bahkali AH, Karunarathna SC, Liu JK, Luangsa‐ard JJ, Lumbsch HT, Maharachchikumbura SSN, McKenzie EHC, Moncalvo JM, Ghobad‐Nejhad M, Nilsson H, Pang KL, Pereira OL, Phillips AJL, Raspé O, Rollins AW, Romero AI, Etayo J, Selçuk F, Stephenson SL, Suetrong S, Taylor JE, Tsui CKM, Vizzini A, Abdel‐Wahab MA, Wen TC, Boonmee S, Dai DQ, Daranagama DA, Dissanayake AJ, Ekanayaka AH, Fryar SC, Hongsanan S, Jayawardena RS, Li WJ, Perera RH, Phookamsak R, de Silva NI, Thambugala KM, Tian Q, Wijayawardene NN, Zhao RL, Zhao Q, Kang JC, Promputtha I (2015) The Faces of fungi database: Fungal names linked with morphology, phylogeny and human impacts. Fungal Diversity 74(1): 3–18. 10.1007/s13225-015-0351-8

[B24] Jeewon R, Hyde KD (2016) Establishing species boundaries and new taxa among fungi: Recommendations to resolve taxonomic ambiguities. Mycosphere: Journal of Fungal Biology 7(11): 1669–1677. 10.5943/mycosphere/7/11/4

[B25] Kalyaanamoorthy S, Minh BQ, Wong TKF, von Haeseler A, Jermiin LS (2017) ModelFinder: Fast model selection for accurate phylogenetic estimates. Nature Methods 14(6): 587–589. 10.1038/nmeth.4285 PMC5453245 28481363

[B26] Kearse M, Moir R, Wilson A, Stones‐Havas S, Cheung M, Sturrock S, Buxton S, Cooper A, Markowitz S, Duran C, Thierer T, Ashton B, Meintjes P, Drummond A (2012) Geneious Basic: An integrated and extendable desktop software platform for the organization and analysis of sequence data. Bioinformatics (Oxford, England) 28(12): 1647–1649. 10.1093/bioinformatics/bts199 PMC3371832 22543367

[B27] Li GJ, Hyde KD, Zhao RL, Sinang H, Abdel-Aziz FA, Abdel-Wahab MA, Alvarado P, Alves-Silva G, Ammirati JF, Ariyawansa HA, Baghela A, Bahkali AH, Beug M, Bhat DJ, Bojantchev D, Boonpratuang T, Bulgakov TS, Camporesi E, Boro MC, Ceska O, Chakraborty D, Chen JJ, Chethana KWT, Chomnunti P, Consiglio G, Cui BK, Dai DQ, Dai YC, Daranagama DA, Das K, Dayarathne MC, De Crop E, De Oliveira RJV, Fragoso de Souza CA, de Souza JI, Dentinger BTM, Dissanayake AJ, Doilom M, Drechsler-Santos ER, Ghobad-Nejhad M, Gilmore SP, Góes-Neto A, Gorczak M, Haitjema CH, Hapuarachchi KK, Hashimoto A, He MQ, Henske JK, Hirayama K, Iribarren MJ, Jayasiri SC, James TY, Jayawardena RS, Jeon SJ, Jerônimo GH, Jesus AL, Jones EBG, Kang JC, Karunarathna SC, Kirk PM, Konta S, Kuhnert E, Langer E, Lee HS, Lee HB, Li WJ, Li XH, Liimatainen K, Lima DX, Lin CG, Liu JK, Liu XZ, Liu ZY, Luangsa-ard JJ, Lücking R, Lumbsch HT, Lumyong S, Leaño EM, Marano AV, Matsumura M, McKenzie EHC, Mongkolsamrit S, Mortimer P, Nguyen TTT, Niskanen T, Norphanphoun C, O’Malley MA, Parnmen S, Pawłowska J, Perera RH, Phookamsak R, Phukhamsakda C, Pires-Zottarelli CLA, Raspé O, Reck MA, Rocha SCO, Santiago ALCM de A, Senanayake IC, Setti L, Shang QJ, Singh SK, Sir EB, Solomon KV, Song J, Srikitikulchai P, Stadler M, Suetrong S, Takahashi H, Takahashi T, Tanaka K, Tang LP, Thambugala KM, Thanakitpipattana D, Theodorou MK, Thongbai B, Thummarukcharoen T, Tian Q, Tibpromma S, Verbeken A, Vizzini A, Vlasák J, Voigt K, Wanasinghe DN, Wang Y, Weerakoon G, Wen HA, Wen TC, Wijayawardene NN, Wongkanoun S, Wrzosek M, Xiao YP, Xu JC, Yan JY, Yang J, Yang SD, Hu Y, Zhang JF, Zhao J, Zhou LW, Peršoh D, Phillips AJL, Maharachchikumbura SSN (2016) Fungal diversity notes 253–366: Taxonomic and phylogenetic contributions to fungal taxa. Fungal Diversity 78: 1–237. 10.1007/s13225-016-0366-9

[B28] Liu YJ, Whelen S, Hall BD (1999) Phylogenetic relationships among ascomycetes: Evidence from an RNA polymerse II subunit. Molecular Biology and Evolution 16(12): 1799–1808. 10.1093/oxfordjournals.molbev.a026092 10605121

[B29] Mattoo AJ, Ghosh A, Nonzom S (2023) *Comoclathris acuminata* (Pleosporaceae, Pleosporales): A new endophytic species from Indian Himalayas. Phytotaxa 589(3): 230–244. 10.11646/phytotaxa.589.3.2

[B30] Miller MA, Pfeiffer W, Schwartz T (2010) Creating the CIPRES Science Gateway for inference of large phylogenetic trees. 2010 Gateway Computing Environments Workshop (GCE). IEEE Computer Society, New Orleans, LA, 1–8. 10.1109/GCE.2010.5676129

[B31] Moral J, Agusti-Brisach C, Perez-Rodriguez M, Xavier C, Raya MC, Rhouma A, Trapero A (2017) Identification of fungal species associated with branch dieback of olive and resistance of table cultivars to *Neofusicoccum mediterraneum* and *Botryosphaeria dothidea*. Plant Disease 101(2): 306–316. 10.1094/PDIS-06-16-0806-RE 30681917

[B32] Nograsek A (1990) Ascomyceten auf Gefässpflanzen der Polsterseggenrasen in den Ostalpen. Bibliotheca Mycologica 133: 271 pp. [+ 5 pl.]

[B33] Pande AK (1980) Ascomycetes of Western India. VIII. 236–241. Sydowia: 33.

[B34] Patil MS, Pawar AB (1987) Studies on foliicolous ascomycetes – IV. Indian Phytopathology 39(4): 515–520.

[B35] Patil SD, Ramesh C (1987) Notes on some fungi of Pleosporaceae (Loculoascomycetes) from Maharashtra (India). Nippon Kingakkai Kaiho 28(3): 229–236.

[B36] Petrak F (1938) List of new species and varieties of fungi, new combinations and new names published. Index of Fungi 5: 171–294.

[B37] Rambaut A (2012) FigTree version 1. 4. University of Edinburgh, Edinburgh.

[B38] Rannala B, Yang Z (1996) Probability distribution of molecular evolutionary trees: A new method of phylogenetic inference. Journal of Molecular Evolution 43: 304–311. 10.1007/BF02338839 8703097

[B39] Rathnayaka AR, Tennakoon DS, Jones GE, Wanasinghe DN, Bhat DJ, Priyashantha AKH, Stephenson SL, Tibpromma S, Karunarathna SC (2024) Significance of precise documentation of hosts and geospatial data of fungal collections, with an emphasis on plant-associated fungi. New Zealand Journal of Botany: 1–28. 10.1080/0028825X.2024.2381734

[B40] Richard MD, Lippmann RP (1991) Neural network classifiers estimate Bayesian a posteriori probabilities. Neural Computation 3: 461–483. 10.1162/neco.1991.3.4.461 31167331

[B41] Ronquist F, Teslenko M, Van Der Mark P, Ayres DL, Darling A, Höhna S, Larget B, Liu L, Suchard MA, Huelsenbeck JP (2012) MrBayes 3.2: Efficient Bayesian phylogenetic inference and model choice across a large model space. Systematic Biology 61(3): 539–542. 10.1093/sysbio/sys029 PMC3329765 22357727

[B42] Senanayake IC, Rathnayaka AR, Marasinghe DS, Calabon MS, Gentekaki E, Lee HB, Hurdeal VG, Pem D, Dissanayake LS, Wijesinghe SN, Bundhun D, Nguyen TT, Goonasekara ID, Abeywickrama PD, Bhunjun CS, Jayawardena RS, Wanasinghe DN, Jeewon R, Bhat DJ, Xiang MM (2020) Morphological approaches in studying fungi, collection examination isolation sporulation and preservation. Mycosphere: Journal of Fungal Biology 11(1): 2678–2754. 10.5943/mycosphere/11/1/20

[B43] Shoemaker RA, Babcock CE (1992) Applanodictyosporous Pleosporales: *Clathrospora*, *Comoclathris*, *Graphyllium*, *Macrospora*, and *Platysporoides*. Canadian Journal of Botany 70(8): 1617–1658. 10.1139/b92-204

[B44] Stamatakis A (2014) RAxML version 8, a tool for phylogenetic analysis and post‐analysis of large phylogenies. Bioinformatics (Oxford, England) 30(9): 1312–1313. 10.1093/bioinformatics/btu033 PMC3998144 24451623

[B45] Stamatakis A, Hoover P, Rougemont J (2008) A rapid bootstrap algorithm for the RAxML web servers. Systematic Biology 57(5): 758–771. 10.1080/10635150802429642 18853362

[B46] Su YY, Sun X, Guo LD (2011) Seasonality and host preference of arbuscular mycorrhizal fungi of five plant species in the inner Mongolia steppe, China. Brazilian Journal of Microbiology: Publication of the Brazilian Society for Microbiology 42: 57–65. 10.1590/S1517-83822011000100008 PMC3768904 24031605

[B47] Thambugala KM, Wanasinghe DN, Phillips AJL, Camporesi E, Bulgakov TS, Phukhamsakda C, Ariyawansa HA, Goonasekara ID, Phookamsak R, Dissanayake A, Tennakoon DS, Tibpromma S, Chen YY, Liu ZY, Hyde KD (2017) Mycosphere notes 1–50: Grass (Poaceae) inhabiting Dothideomycetes. Mycosphere: Journal of Fungal Biology 8: 697–79. 10.5943/mycosphere/8/4/13

[B48] Tibpromma S, Promputtha I, Phookamsak R, Boonmee S, Hydem KD (2015) Phylogeny and morphology of *Premilcurensis* gen. nov. (Pleosporales) from stems of *Senecio* in Italy. Phytotaxa 236(1): 40. 10.11646/phytotaxa.236.1.3

[B49] Vilgalys R, Hester M (1990) Rapid genetic identification and mapping of enzymatically amplified ribosomal DNA from several *Cryptococcus* species. Journal of Bacteriology 172(8): 4238–4246. 10.1128/jb.172.8.4238-4246.1990 PMC213247 2376561

[B50] Vu D, Groenewald M, Vries MD, Gehrmann T, Stielow B, Eberhardt U, Al-Hatmi A, Groenewald JZ, Cardinali G, Houbraken J (2019) Large-scale generation and analysis of filamentous fungal DNA barcodes boosts coverage for kingdom fungi and reveals thresholds for fungal species and higher taxon delimitation. Studies in Mycology 92: 135–154. 10.1016/j.simyco.2018.05.001 PMC6020082 29955203

[B51] Wanasinghe DN, Gareth JEB, Camporesi E, Hyde KD (2015) A new species of the genus *Comoclathris* (Pleosporaceae). Journal of Fungal research 13: 260–268.

[B52] Wanasinghe DN, Phukhamsakda C, Hyde KD, Jeewon R, Lee HB, Gareth Jones EBG, Tibpromma S, Tennakoon DS, Dissanayake AJ, Jayasiri SC, Gafforov Y, Camporesi E, Bulgakov TS, Ekanayake AH, Perera RH, Samarakoon MC, Goonasekara ID, Mapook A, Li WJ, Senanayake IC, Li J, Norphanphoun C, Doilom M, Bahkali AH, Xu J, Mortimer PE, Tibell L, Tibell S, Karunarathna SC (2018) Fungal diversity notes 709–839: Taxonomic and phylogenetic contributions to fungal taxa with an emphasis on fungi on Rosaceae. Fungal Diversity 89(1): 1–236. 10.1007/s13225-018-0395-7

[B53] Wang Q, Bao Y, Nan J, Xu D (2020) AM fungal diversity and its impact across three types of mid-temperate steppe in Inner Mongolia, China. Mycorrhiza 30(1): 97–108. 10.1007/s00572-019-00926-x 31832763

[B54] Wang YL, Zhang X, Xu Y, Babalola BJ, Xiang SM, Zhao YL, Fan YJ (2021) Fungal diversity and community assembly of ectomycorrhizal fungi associated with five pine species in Inner Mongolia, China. Frontiers in Microbiology 12: 646821. 10.3389/fmicb.2021.646821 PMC8008119 33796093

[B55] Wang R, Meng Z, Gao Y, Wu Z (2024) Herbaceous plant diversity and soil physicochemical properties under different artificial forests in the Bulianta core mine, Inner Mongolia, China. Forests (19994907) 15(10). 10.3390/f15101713

[B56] Webster J, Lucas MT (1959) Observations on British species of *Pleospora*. I. Transactions of the British Mycological Society 42(3): 332–342. 10.1016/S0007-1536(56)80042-9

[B57] White TJ, Bruns T, Lee SJWT, Taylor JL (1990) Amplification and direct sequencing of fungal ribosomal RNA genes for phylogenetics. In: Innis MA, Gelfand DH, Sninsky JJ, White TJ (Eds) PCR protocols, a guide to methods and applications. Academic Press, San Diego, CA, 315–322. 10.1016/B978-0-12-372180-8.50042-1

[B58] Woudenberg JHC, Groenewald JZ, Binder M, Crous PW (2013) *Alternaria* redefined. Studies in Mycology 75(1): 171–212. 10.3114/sim0015 PMC3713888 24014900

[B59] Woudenberg JHC, Hanse B, Van Leeuwen GCM, Groenewald JZ, Crous PW (2017) *Stemphylium* revisited. Studies in Mycology 87: 77–103. 10.1016/j.simyco.2017.06.001 PMC5480992 28663603

[B60] Xu R, Su W, Wang Y, Tian S, Li Y, Phukhamsakda C (2024) Morphological characteristics and phylogenetic evidence reveal two new species and the first report of *Comoclathris* (Pleosporaceae, Pleosporales) on dicotyledonous plants from China. MycoKeys 101: 95. 10.3897/mycokeys.101.113040 PMC10799302 38250088

[B61] Yan X, Wei D, Yang J, Yao W, Tian S (2025) Monitoring Temperate Typical Steppe Degradation in Inner Mongolia: Integrating Ecosystem Structure and Function. Sustainability 17(20): 9015. 10.3390/su17209015

[B62] Yang Z, Zhang B, Qu Z, Song Z, Pan X, Zhao C, Ma H (2022) Two new species of *Diatrype* (Xylariales, Ascomycota) with polysporous asci from China. Diversity 14(2): 149. 10.3390/d14020149

[B63] Zeng XY, Tan TJ, Tian FH, Wang Y, Wen TC (2023) OFPT: A one-stop software for fungal phylogeny. Mycosphere: Journal of Fungal Biology 14(1): 1730–1741. 10.5943/mycosphere/14/1/20

[B64] Zhang Y, Crous PW, Schoch CL, Hyde KD (2012) Pleosporales. Fungal Diversity 53(1): 1–221. 10.1007/s13225-011-0117-x PMC3477819 23097638

[B65] Zhaxybayeva O, Gogarten JP (2002) Bootstrap, Bayesian probability and maximum likelihood mapping: Exploring new tools for comparative genome analyses. BMC Genomics 3: 4. 10.1186/1471-2164-3-4 PMC100357 11918828

